# A structural equation model analysis of the relationship between maternal fear of childbirth and expectant fathers’ fear of childbirth: The mediating role of fathers’ depression, anxiety, and stress

**DOI:** 10.1002/brb3.2802

**Published:** 2022-10-26

**Authors:** Seyedeh Fatemeh Ghaffari, Forouzan Elyasi, Roya Nikbakht, Zohreh Shahhosseini

**Affiliations:** ^1^ Student Research Committee Mazandaran University of Medical Sciences Sari Iran; ^2^ Sexual and Reproductive Health Research Center, Psychiatry and Behavioral Sciences Research Center, Addiction Institute Mazandaran University of Medical Sciences Sari Iran; ^3^ Department of Biostatistics and Epidemiology, Faculty of Health Golestan University of Medical Sciences Gorgan Iran; ^4^ Sexual and Reproductive Health Research Center Mazandaran University of Medical Sciences Sari Iran

**Keywords:** maternal fear of childbirth, paternal fear of childbirth, pregnancy, structural equation model

## Abstract

**Introduction:**

Some fathers experience traumatic and unpleasant feelings such as fear of childbirth during pregnancy and childbirth. This study aimed to determine the mediating role of the expectant fathers’ depression, anxiety, and stress in the relationship between maternal fear of childbirth and paternal fear of childbirth.

**Methods:**

In this cross‐sectional study, using a two‐stage sampling method, 502 expectant Iranian fathers and their wives in the second half of pregnancy were recruited. The participants completed self‐administered questionnaires, including the fathers’ fear of childbirth scale, the Wijma delivery expectancy/experience questionnaire, and the depression, anxiety, and stress scale‐21. To analyze the data, structural equation modeling was employed in the Amos software version 24.

**Results:**

Results indicated an acceptable fit of the model to the data. Maternal fear of childbirth was associated with paternal fear of childbirth, directly (*β* = 0.23, *p* = .046) and indirectly through the mediator of paternal depression, anxiety, and stress (*β* = .17, *p* = .007). The expectant fathers’ stress had a greater impact on their fear of childbirth.

**Conclusions:**

By considering the role of maternal fear of childbirth as well as expectant fathers’ depression, anxiety, and stress on paternal fear of childbirth, it seems this study has some practical implications for improving the fathers’ psychological well‐being.

## Graphical Abstract

1‐Like mothers, fathers may also suffer from fear of childbirth.

2‐Fathers ‘fear of childbirth is influenced by mothers’ fear of childbirth.

3‐Psychological factors such as stress affect the fear of childbirth in fathers.

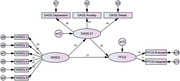

## 1 INTRODUCTION

Pregnancy and childbirth are physiological processes that are commonly referred to as pleasurable periods of life (Eriksson, Jansson, et al., 2006; Nilsson & Lundgren, 2009). For some women and their husbands, however, a range of unpleasant feelings such as fear are experienced in this period (Masoumi & Elyasi, 2021; Meleis et al., 2000; Schumacher et al., 2008). Becoming a father, even when desired and planned, can be a difficult transition for some men, negatively affecting their mental health and leading to stress, anxiety, and depression (Leach et al., 2016; Philpott et al., 2017). Reducing expectant fathers’ self‐confidence, unpleasant experience of pregnancy and childbirth, and post‐traumatic stress disorder may be related to paternal fear of childbirth (Bergström et al., 2013; Hanson et al., 2009; Hildingsson, Johansson, et al., 2014).

Fear of childbirth is a negative view that begins in the prenatal period and continues during and after childbirth is classified as mild to severe (Demšar et al., 2018; Erkaya et al., 2017). Meanwhile, mild fear of childbirth is attributed to the normal processes of pregnancy and childbirth, its moderate level is difficult to manage but does not adversely affect one's current mental health. Severe fear affects an expectant fathers’ mental health and significantly disrupts his daily life as well as paternal‐fetal attachment (Ekelin & Crang‐Svalenius, 2004). Fear of childbirth in fathers can be due to various factors such as fear of the labor process (Eriksson, Westman, et al., 2006), harm to the health of his wife and child (Eriksson, Westman, et al., 2006; Hanson et al., 2009), his wife's suffering (Hanson et al., 2009; Jean Greer, 2014), inability to support his wife (Jean Greer, 2014), financial constraints (Hanson et al., 2009), and impairment in the quality of the future marital relationship (Hanson et al., 2009). Fear of childbirth has been reported in 11%–37% of fathers (Bergström et al., 2009; Eriksson et al., 2005; Hildingsson, Johansson, et al., 2014), and it is reported that about 13% of them experience the severe fear of childbirth (Eriksson et al., 2005; Hildingsson, Johansson, et al., 2014; Moran et al., 2021).

To the best of our knowledge, the existing literature was mainly focused on the fear of childbirth in mothers, and our knowledge about the paternal fear of childbirth and its related factors is limited. According to the International Conference on Population and Development agenda, the role of fathers in the various aspects of reproductive health, including the birth process, is emphasized (Johansson et al., 2021). Hence, by considering the role of men's participation in family health, and by attention to the importance of empowering men to take responsibility during pregnancy and childbirth, paying attention to the expectant fathers’ mental health is a crucial subject. Therefore, this study was designed to determine the mediating role of the expectant fathers’ depression, anxiety, and stress in the relationship between maternal fear of childbirth and paternal fear of childbirth.

## 2 MATERIALS AND METHODS

### 2.1 Study design

In this cross‐sectional study, 502 expectant fathers were recruited from May 2019 to August 2020.

### 2.2 Inclusion and exclusion criteria

Literate expectant fathers whose spouses were at ≥20 weeks of pregnancy were recruited.

### 2.3 Sample size

According to Hildingsson et al.’s study, the prevalence of fear of childbirth equal to 13.6%, *d* = 0.03, confidence level = 95%, and study power = 80%, the sample size included 502 fathers (Hildingsson, Johansson, et al., 2014).

### 2.4 Sampling

Sampling was conducted in all public primary health care centers (*n* = 20) affiliated to Mazandaran University of Medical Sciences (MAZUMS), Iran. In the Iranian health care system, all pregnant mothers receive routine prenatal care in public primary health care centers during pregnancy. Sampling was conducted in two stages. First, according to the number of pregnant mothers aged ≥20 weeks of pregnancy in each primary health care center, the required sample size in each center was identified, then they were selected based on the convenient method. After making a phone call to the selected mothers and introducing a summary of the project, interested mothers were asked to discuss the project with their spouses. Finally, the anonymous self‐ administrated questionnaires were completed by the expectant father and his wife at one of the prenatal care visits.

### 2.5 Measurements

#### 2.5.1 Depression, anxiety, and stress scale‐21

The short version of this instrument is a 21‐item scale with three subscales including depression, anxiety, and stress, each with seven items (Sahebi et al., 2005). Scoring is based on the Likert scale from zero to three with the score ranging from zero to 21 in each subscale. Cronbach's alpha of 0.94, 0.87, and 0.91 for the depression, anxiety, and stress subscales, respectively, shows an acceptable internal consistency of this scale (Antony et al., 1998).

#### 2.5.2 Fathers’ fear of childbirth scale

This scale was developed by Ghaffari et al. in a sample of 433 Iranian fathers (Ghaffari et al., 2021). Fathers’ fear of childbirth scale (FFCS) includes 17 items with two subscales entitled fear of childbirth (12 items) and fear of hospital (five items). The FFCS was scored on a five‐point Likert scale from one to five. Therefore, the total score of the scale varies from 17 to 85 and the higher score indicates greater fear of childbirth. The score of 17–35 shows low fear, 36–54 moderate fear, and 55 and above indicated severe fear. The FFCS had the desired validity and reliability. The factors explained 50.82% of the total variance. The reliability of the scale has been obtained as Cronbach's alpha of 0.84 (Ghaffari et al., 2021).

#### 2.5.3 Wijma delivery expectancy/experience questionnaire

This instrument was developed by Wijma et al. in 1998 consisting of 33 items in a six‐point range, from zero to five points (Wijma et al., 1998). The overall score ranges from zero to 165; the higher score indicates greater fear of childbirth. The reliability of the questionnaire by split‐half testing and Cronbach's alpha is 0.89 and 0.93, respectively (Mortazavi, 2017).

### 2.6 Ethical consideration

The study was approved by the Iranian National Committee for Ethics in Biomedical Research, Ethical Code: IR.MAZUMS.REC.1398.1412. All participants signed informed consent forms that met the Declaration of Helsinki guidelines; there was no financial compensation.

### 2.7 Data analysis

Employing SPSS version 21, we calculated the mean, standard deviation, and Cronbach's alpha to depict the numerical variables, while frequency and percentage were used for describing the categorical variables. Pearson correlation coefficient was employed to assess the relationship between numerical variables. Skewness and Kortusis have been reported to test the normality of variables. The alignment hypothesis was also tested with tolerance coefficient and variance inflation factor. Amos version 24 was employed to analyze the relationship between the variables. The dependent variable was fathers’ fear of childbirth, the independent variable was maternal fear of childbirth, and the fathers’ depression, anxiety, and stress were set as mediating variables. The basis of data analysis was the structural equation modeling (SEM) with a significance level of 0.05.

## 3 RESULTS

The data of 502 participants who had completed all the relevant questionnaires were analyzed. The mean ages of the expectant fathers, the pregnant mothers, age and their gestational age were 33.29 ± 6.33, 31.20 ± 5.55 years, and 29.36 ± 4.93 weeks, respectively. The prevalence of low, moderate, and severe fear of childbirth in fathers was 15.74%, 42.03%, and 42.23%, respectively. The mean score and standard deviation of the father's and mother's fear of childbirth were 50.11 ± 13.65 and 58.11 ± 24.32, respectively. Most fathers (82.60%) had a diploma or higher degree education, 71.65% were self‐employed, and 56.60% had no children.

Descriptive statistics are shown in Table 1. As it is illustrated, there is a significant correlation between the variables at the .01 significance level. The results reported in Table 2 confirm the normality of the variables. Also, according to the variance inflation factor and tolerance coefficient, there is no alignment between variables.

**TABLE 1:** The descriptive statistics for independent, mediator, and dependent variables

Variables	Descriptive statistics		Correlations among variables									
	Mean	SD	1	2	3	4	5	6	7	8	9	10
1. DASS‐Depression	4.96	0.30	1									
2. DASS‐Anxiety	5.61	0.33	0.76**	1								
3. DASS‐Stress	9.80	0.39	0.72**	0.74**	1							
4. W‐DEQ‐LOE	17.19	0.44	0.32**	0.30**	0.30**	1						
5. W‐DEQ‐LOP	5.93	0.19	0.28**	0.29**	0.26**	0.58**	1					
6. W‐DEQ‐loneliness	13.69	0.38	0.35**	0.39**	0.31**	0.36**	0.32**	1				
7. W‐DEQ‐Fear	14.15	0.25	0.41**	0.41**	0.37**	0.43**	0.34**	0.60**	1			
8. W‐DEQ‐CC	2.03	0.11	0.26**	0.27**	0.24**	0.26**	0.28**	0.25**	0.37**	1		
9. W‐DEQ‐CLC	5.76	0.15	0.31**	0.34**	0.31**	0.40**	0.43**	0.57**	0.53**	0.28**	1	
10. FFCS	50.11	13.65	0.28**	0.25**	0.40**	0.22**	0.18**	0.26**	0.24**	0.12**	0.23**	1

Abbreviations: CC, concern for the child; CLC, concern about losing control; DASS, depression, anxiety, and stress scale; FFCS, fathers’ fear of childbirth scale; LOE, lack of efficacy; LOP, lack of positive antiception; W‐DEQ, Wijma delivery expectancy/experience questionnaire.

** Correlation is significant at the 0.01 level (2‐tailed).

**TABLE 2:** Skewness, Kortusis, tolerance coefficient, and variance inflation factor of variables

Variables	Skewness	Kortusis	Tolerance	Variance inflation factor
FFCS	−0.32	−0.52	–	–
W‐DEQ‐LOE	0.67	0.01	0.56	1.70
W‐DEQ‐LOP	0.78	0.24	0.60	1.66
W‐DEQ‐Loneliness	0.56	−0.35	0.54	1.85
W‐DEQ‐Fear	−0.26	−0.53	0.51	1.96
W‐DEQ‐CC	1.28	0.80	0.82	1.21
W‐DEQ‐CLC	0.16	−0.64	0.57	1.77
DASS‐Depression	1.99	3.10	0.36	2.81
DASS‐Anxiety	1.75	2.75	0.34	2.97
DASS‐Stress	1.10	0.67	0.39	2.56

Abbreviations: CC, concern for the child; CLC, concern about losing control; DASS, depression, anxiety, and stress scale; FFCS, fathers’ fear of childbirth scale; LOE, lack of efficacy; LOP, lack of positive antiception; W‐DEQ, Wijma delivery expectancy/experience questionnaire.

**FIGURE 1:**
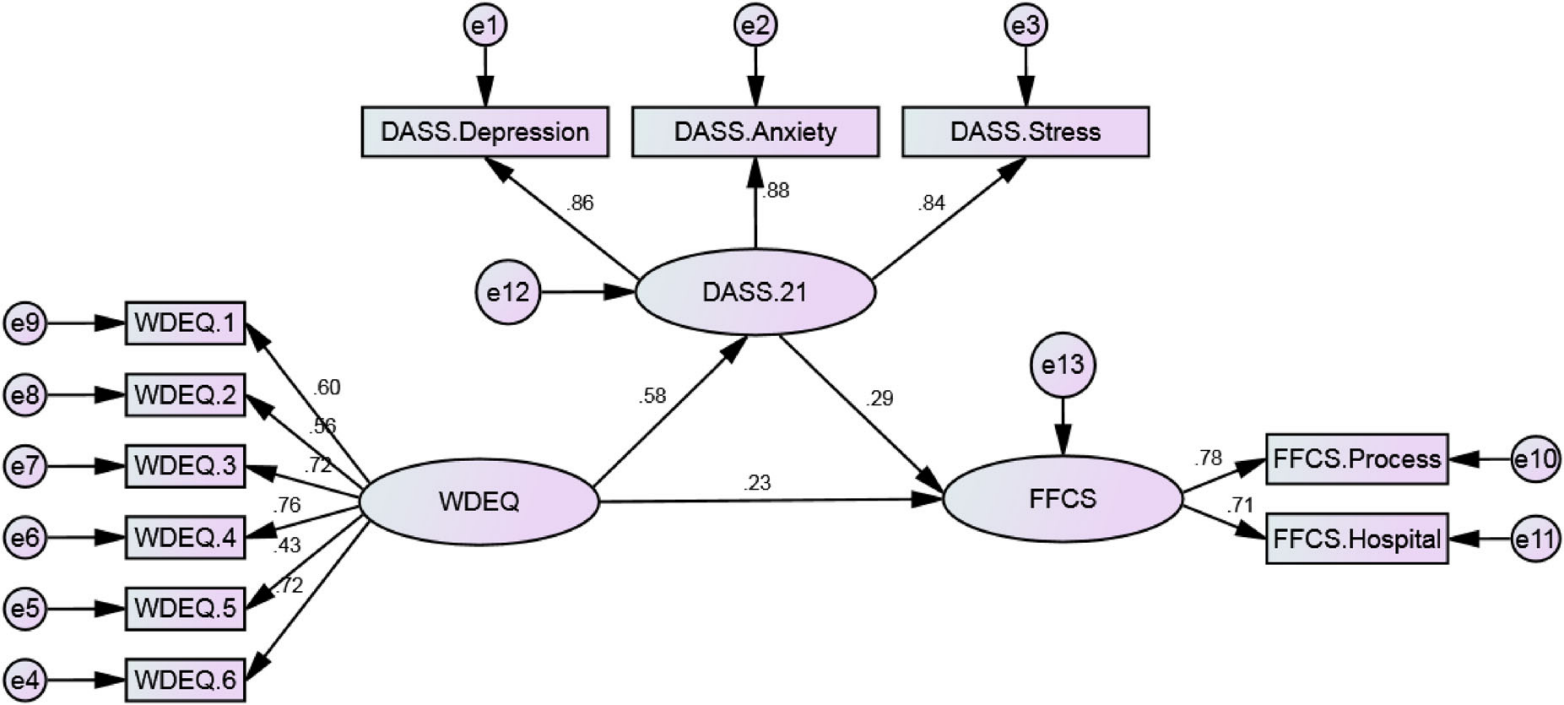
Research model and its parameters using standardized data

The major purpose of the research measurement model was to determine whether the structural model of the mediating role of paternal depression, anxiety, and stress explains an appropriate fit concerning maternal fear of childbirth and paternal fear of childbirth relationships in the expectant fathers. The initial model was examined by structural equations using maximum likelihood estimation. In the final model, the quantity of *χ* ^2^/*df* was equal to 4.48, the comparative fit index (CFI) was 0.94, the parsimony comparative fit index (PCFI) was 0.70, and the root mean square error of approximation (RMSEA) was obtained 0.08 (the accessibility cut points for each model fit indexes: *χ* ^2^/*df* < 5, CFI > 0.9, PCFI > 0.5 and RMSEA < 0.05), indicating the acceptability of the goodness of fit model index (Schreiber et al., 2006). Figure 1 demonstrates the research final measurement model and its parameters.

The results reported in Table 3 display the direct and indirect standardized effects of the variables and the significance level of this correlation based on the bias‐corrected percentile method. The results showed that paternal depression, anxiety, and stress (measured by depression, anxiety, and stress scale‐21 [DASS‐21]) were directly associated with paternal fear of childbirth measured by FFCS (*β* = 0.29, *p* = .009). Also, maternal fear of childbirth (measured by Wijma delivery expectancy/experience questionnaire [W‐DEQ]) has a statistically significant relationship with paternal fear of childbirth, directly (*β* = 0.23, *p* = .046) and indirectly through the mediator of paternal depression, anxiety, and stress (*β* = 0.58 × 0.29 = 0.17, *p* = .007). To determine the subscale of the DASS‐21 variable that has a greater effect on fathers’ fear of childbirth, the linear regression model was used and the standardized coefficients for the depression, anxiety, and stress were 0.28, 0.25, and 0.40, respectively.

**TABLE 3:** Results for standardized effects of variables

Variables	Total effects	Direct effect	Indirect effect
W‐DEQ → DASS‐21	0.58	0.58	–
95% BC^a^ (*p*‐value^b^)	0.49, 0.66 (0.016)	0.49, 0.66 (0.016)	–
W‐DEQ → FFCS	0.39	0.23	0.17
95% BC (*p*‐value)	0.23, 0.48 (0.030)	0.01, 0.35 (0.046)	0.09, 0.26 (0.007)
DASS‐21→ FFCS	0.29	0.29	–
95% BC (*p*‐value)	0.14, 0.43(0.009)	0.14, 0.43 (0.009)	–
*χ* ^2^/*df* = 4.48, CFI = 0.94, RMSEA = 0.08, PCFI = 0.70			

Abbreviations: CFI, comparative fit index; DASS‐21, depression, anxiety, and stress scale‐21; PCFI, parsimony comparative fit index; RMSEA, root mean square error of approximation; W‐DEQ, Wijma delivery expectancy/experience questionnaire.

^a^ BC: Bias‐corrected confidence Interval using Bootstrap.

^b^ Two‐tailed significance BC.

## 4 DISCUSSION

Fathers’ fear is a subject that has been little studied, and due to the increasing participation of expectant fathers during pregnancy and childbirth, their mental health is an issue of great importance. This study aimed to determine the relationship between maternal fear of childbirth and expectant fathers’ fear of childbirth by considering the mediating role of fathers’ depression, anxiety, and stress via the structural equation modeling approach. Such analyses would have much more far‐reaching implications, given that this analysis give insight into which variables drive the changes in fathers’ fear of childbirth, it may hint at important ports of entry for interventions. Although the prevalence and severity of paternal fear of childbirth are largely depended on the various tools to measure the expectant fathers’ fear in the different sociocultural societies, its severe form in 15.7% of the Iranian expectant fathers in line with the prevalence of 13% in the Swedish fathers means that fear of childbirth is a global health concern that must be taken into account (Eriksson et al., 2005; Hildingsson, Johansson, et al., 2014).

Our findings about the relationship of maternal fear of childbirth (directly and indirectly) with paternal fears of childbirth are in accordance with previous studies which demonstrate couples’ mental health is affected by each other (Barooj‐Kiakalaee et al., 2022; Jamali et al., 2018; Moran et al., 2021; Serçekuş et al., 2020). One of the important reasons for this relationship can be that fathers do not have access to reliable sources of information about pregnancy and childbirth, so they receive most of their information in this area from their spouse (Kunjappy‐Clifton, 2008; Masoumi & Elyasi, 2021). Thus, negative and horror stories cause fear in both (Fisher et al., 2006). This finding broadly supports the idea that couples’ health promotion programs should be implemented in pairs (Jamali et al., 2018). Factors such as fathers’ lack of knowledge, cultural barriers, job problems, women's environment in health centers, inappropriate behavior of health care workers, customary factors, and financial problems are among the barriers to fathers’ presence in pregnancy programs (Mortazavi & Mirzaii, 2012). By reducing these barriers and the participation of fathers in promoting reproductive health, a significant level of fear of each couple can be reduced (Jamali et al., 2018; Serçekuş et al., 2020). As a result, improving the health of couples affects each other and ultimately promotes a positive delivery experience (Hildingsson, Johansson, et al., 2014; Serçekuş et al., 2020).

In accordance with a previous study on this issue (Hildingsson, Haines, et al., 2014), SEM analysis showed that paternal stress was more related to fathers' fear of childbirth than paternal anxiety and depression. Feelings of stress and anxiety may arise from the unknown, uncontrollable, and unavoidable condition of the forthcoming delivery (Bewley & Cockburn, 2002). For some people, this condition is psychologically very stressful, which causes fear of childbirth (Ganapathy, 2015).

### 4.1 Strengths and limitations

One of the strengths of the present study is that the present study has been one of the few studies that have been done in the field of fathers ' fear of childbirth. By dint of SEM analysis, we could determine the simultaneous effect of several variables on the dependent variable. It can process variables (latent variables) that are not directly observed and can be a measurement model more flexible than other statistical methods.

The results must be interpreted in light of some limitations. Self‐reporting fear, depression, anxiety, and stress questionnaires are less reliable compared to clinical findings. Another limitation of the study is that fathers are reluctant to express fear and it is difficult for them to talk about fear (Eriksson, Westman, et al., 2006). This issue in patriarchal societies such as Iran, where fathers may see the expression of their feelings in the form of fear as an indicator of their patriarchal personality weakness, may limit our interpretation of the findings. In this regard, we tried to assure the participants that their answers will be confidential. We also encouraged them to answer the questionnaires honestly. This study used a convenience sampling technique, which may limit the external validity and therefore threaten the generalizability of the results. Therefore, it is suggested to use other sampling methods such as random sampling methods in future studies.

## 5 CONCLUSION

Improving the fear of childbirth is a good strategy to improve family health. Therefore, it is important to pay attention to the role of effective factors and main mediators in formulating these strategies. The present study confirmed the mediating role of depression, anxiety, and stress of expectant fathers in the relationship between mother and father fear of childbirth. It is recommended to conduct interventional studies to investigate the role of these variables on fathers' fears of childbirth in longitudinal studies.

## AUTHOR CONTRIBUTIONS

Seyedeh Fatemeh Gaffari, Zohreh Shahhosseini, and Forouzan Elyasi contributed to the design of the study. Seyedeh Fatemeh Gaffari contributed to data collection. Roya Nikbakht contributed to the data analysis. Seyedeh Fatemeh Gaffari and Zohreh Shahhosseini wrote the first draft of this manuscript. All authors read and approved the final manuscript.

## CONFLICT OF INTEREST

The authors declare no conflict of interest.

### PEER REVIEW

The peer review history for this article is available at: https://publons.com/publon/10.1002/brb3.2802.

## Data Availability

The data that support the findings of this study are available from the corresponding author upon reasonable request.
